# Effectiveness of Duloxetine versus Other Therapeutic Modalities in Patients with Diabetic Neuropathic Pain: A Systematic Review and Meta-Analysis

**DOI:** 10.3390/ph17070856

**Published:** 2024-06-28

**Authors:** Juan José Valenzuela-Fuenzalida, Michelle López-Chaparro, Marisol Barahona-Vásquez, Javiera Campos-Valdes, Javiera Cordero Gonzalez, Pablo Nova-Baeza, Mathias Orellana-Donoso, Alejandra Suazo-Santibañez, Gustavo Oyanedel-Amaro, Héctor Gutiérrez Espinoza

**Affiliations:** 1 Departamento de Morfología, Facultad de Medicina, Universidad Andrés Bello, Santiago 8370146, Chile; juan.kine.2015@gmail.com (J.J.V.-F.); michelle.lopch@gmail.com (M.L.-C.); marisolvbarahona@gmail.com (M.B.-V.); javiera.campos@uandresbello.edu (J.C.-V.); javiera.cordero@gmail.com (J.C.G.); pablo.nova@usach.cl (P.N.-B.); miorellanadonoso@gmail.com (M.O.-D.); 2 Departamento de Ciencias Química y Biológicas, Facultad de Ciencias de la Salud, Universidad Bernardo O’Higgins, Santiago 8370993, Chile; 3 Escuela de Medicina, Universidad Finis Terrae, Santiago 7501015, Chile; 4 Faculty of Health and Social Sciences, Universidad de Las Américas, Santiago 8370040, Chile; alej.suazo@gmail.com; 5 Facultad de Ciencias de la Salud, Universidad Autónoma de Chile, Santiago 7501019, Chile; g.oyanedelamaro@gmail.com; 6 One Health Research Group, Universidad de las Americas, Quito 170124, Ecuador

**Keywords:** diabetic neuropathy, duloxetine therapy, neuropathic pain, type 1 diabetes mellitus, type 2 diabetes mellitus

## Abstract

**Objectives:** Diabetic peripheral neuropathy (DPN) is a chronic complication of diabetes mellitus (DM) with symptoms like intense pain and impaired quality of life. This condition has no treatment; instead, the pain is managed with various antidepressants, including duloxetine. The aim of this study is to analyze the evidence on the efficacy of duloxetine in the management of DPN. **Methods:** A systematic search in different databases was conducted using the keywords “diabetic neuropathy”, “duloxetine therapy”, “neuropathic pain”, and “Diabetes Mellitus”. Finally, eight studies were included in this meta-analysis. **Results:** All articles comparing duloxetine at different doses vs. a placebo reported significant differences in favor of duloxetine on pain scales like 24 h Average Pain Severity (standardized mean difference [SMD] = −1.06, confidence interval [CI] = −1.09 to −1.03, and *p* < 0.00001) and BPI Severity (SMD = −0.70, CI = −0.72 to −0.68, and *p* < 0.00001), among others. A total of 75% of the meta-analyses of studies comparing duloxetine at different doses showed a tendency in favor of the 120 mg/d dose. There were significant differences in favor of duloxetine when compared to routine care on the Euro Quality of Life (SMD = −0.04, CI = −0.04 to −0.03, and *p* < 0.00001) and SF-36 Survey (SMD = −5.86, CI = −6.28 to −5.44, and *p* < 0.00001) scales. There were no significant differences on the visual analog scale (VAS) when comparing duloxetine and gabapentin. **Conclusions:** Duloxetine appears to be effective in the management of DPN in different pain, symptom improvement, and quality of life scales.

## 1. Introduction

Diabetes Mellitus (DM) is a chronic metabolic disease characterized by elevated glycemia levels [1]. This health condition has reached significant proportions worldwide, with an estimated prevalence of 2.8% of the total population, which is expected to reach 4.4% or 336 million people by 2030 [2]. There are two types of DM, the most common being type 2 DM, where the body becomes resistant to insulin or does not produce adequate insulin. On the other hand, type 1 DM is when the person is completely dependent on insulin because the pancreas produces insufficient or no insulin. High glycemia can lead to more serious complications such as kidney damage and heart, vascular, eye, and nerve disorders [3,4,5,6,7].

Among the complications associated with DM, neuropathic pain (NP) is one of the most frequent and debilitating. NP is a condition associated with peripheral neuropathy, in which damage to nerve fibers produces alterations in the transmission of signals generating pain [7]. Peripheral neuropathy is present in 50% of diabetic patients, and 16% to 26% go on to develop diabetic peripheral neuropathy (DPN) as a chronic complication of the disease. Chronic DPN is associated with pain of varying severity, with symptoms such as tingling, burning, sharp pains, cramping, extreme sensitivity to touch, numbness, and a significant decrease in quality of life [8,9]. The impact on daily life is significant, requiring a comprehensive approach to diagnosis and management [9].

Due to the lack of a treatment for DPN, various drugs and therapies have been investigated and shown to be effective in pain control [10,11,12]. Conventional treatment has included analgesics, anticonvulsants, antidepressants, and optical capsaicin. When pain is mild, these medications usually have optimal results [13,14]. However, if pain becomes disabling, the efficacy of these treatments is limited by the side effects of the drugs [10,11,12].

The drugs most commonly used are antidepressants such as amitriptyline, pregabalin, gabapentin, and duloxetine [15]. Duloxetine is an inhibitor of serotonin and norepinephrine reuptake in the central nervous system, increasing their concentration and availability in the synaptic space, sustaining their antidepressant and analgesic effect, useful in the management of DPN [16,17].

The pharmacological effects of duloxetine have been consistently supported in several controlled clinical trials, positioning it as a valuable option for NP due to DM [16,17,18,19]. Therefore, the aim of this review is to compare the efficacy of duloxetine with other therapies for DPN in order to determine whether it is a valid treatment to decrease pain and improve the quality of life. Duloxetine is generally known as a drug that has a defined use for anxiety syndromes, but in this study, we will also see its use for patients with pain associated with diabetes, where it has not been exhaustively studied.

## 2. Methods

### 2.1. Protocol and Registration

This systematic review and meta-analysis were performed and reported according to the Preferred Reporting Items for Systematic Reviews and Meta-Analyses (PRISMA) statement [20], PROSPERO ID: CRD42024508135.

### 2.2. Literature Search

The literature search process described in the provided text involves a systematic search of electronic databases to identify relevant studies for a specific research question. The search covered several databases, including MEDLINE (via PubMed), EMBASE, SCOPUS, the Cochrane Central Register of Controlled Trials, the Cumulative Index to Nursing and Allied Health Literature, and Web of Science databases. The search aimed to identify randomized or controlled clinical trials published in English or Spanish, using keywords such as “diabetic neuropathy”, “duloxetine therapy”, “neuropathic pain”, and “Diabetes Mellitus” in various combinations. The screening of titles and abstracts was conducted independently by two authors, with a third reviewer involved if a consensus could not be reached.

### 2.3. Study Selection

For the studies included in this meta-analysis, the following inclusion criteria were used: patients with NP that were directly associated with type 1 or 2 DM; patients who were administered duloxetine at different doses; that the outcomes to be evaluated were pain, disability, symptom improvement, and quality of life scales; and the type of studies included were clinical trials, randomized clinical trials, and experimental studies. Studies with the following characteristics were excluded: letters to the editor, reports/case series, reviews, or non-human trials; studies that enrolled patients with other diseases; studies that administered therapies other than duloxetine; and studies that did not have a control group as a comparator.

### 2.4. Data Extraction and Quality Assessment

The research team, consisting of authors M.B-V. and M.L-C., conducted an analysis of relevant data for each trial independently. The analysis included various aspects such as the authors and year of publication, type of study and total number of participants, statistical values and main results, geographic region, sex distribution, and intervention dose and type of administration. The methodological quality of the included studies was assessed using the Cochrane RoB tool, which evaluates the risk of bias in 7 domains. These domains include the generation of a randomized sequence, concealment of the randomization sequence, blinding of participants and treatments, blinding of outcome assessment, incomplete results, selective reporting of results, and other sources of bias. Disagreements were resolved through discussion or determined by a third reviewer, J.J.V-F., if consensus could not be reached. The agreement rate between reviewers was calculated using kappa statistics, resulting in substantial agreement with a value of 0.88.

### 2.5. Data Synthesis and Analysis

In the assessment of NP (neuropathic pain), various scales were utilized as continuous outcomes, and the effect size was calculated as the standard mean difference (SMD) using Cohen’s d as the effect size statistic. The scales used for assessment included the 24 h Average Pain Severity score, BPI Severity, BPI Interference, CGI Severity, SF-MPQ Total Score, Euro Quality of Life Questionnaire, SF-36 Survey Bodily Pain, and VAS. The effect sizes were categorized as trivial (<0.2), small (0.2–0.5), medium (0.6–0.8), or large (>0.8). Additionally, depending on the heterogeneity of the data, the Hartung–Knapp–Sidik–Jonkman random effect or Mantel–Haenszel fixed effect methods were employed to quantify the pooled effect size of the studies included. The effect sizes were presented as SMD, with their respective 95% confidence intervals (CIs) in the range between 2 and −2. The heterogeneity of results was evaluated using the I2 statistic, which considers 0% to 40% as “may not be important”, 30% to 60% as “moderate”, 50% to 90% as “substantial”, and 75% to 100% as “considerable” heterogeneity. Visual inspection was also conducted to detect overlapping CIs in the forest plots as well as the corresponding *p* values. The meta-analysis was performed using RevMan 5.4.

### 2.6. Rating the Quality of Evidence 

The synthesis and quality of evidence for each outcome were assessed using the grading of recommendation, assessment, development, and evaluation (GRADE). The quality of the evidence was classified into 4 categories: high, moderate, low, and very low [21,22]. We used the GRADE profiler to import the data from RevMan 5.4 to create a “summary of findings” table, which can be found in Supplementary Table S3.

## 3. Results

### 3.1. Study Selection

**Figure 1:**
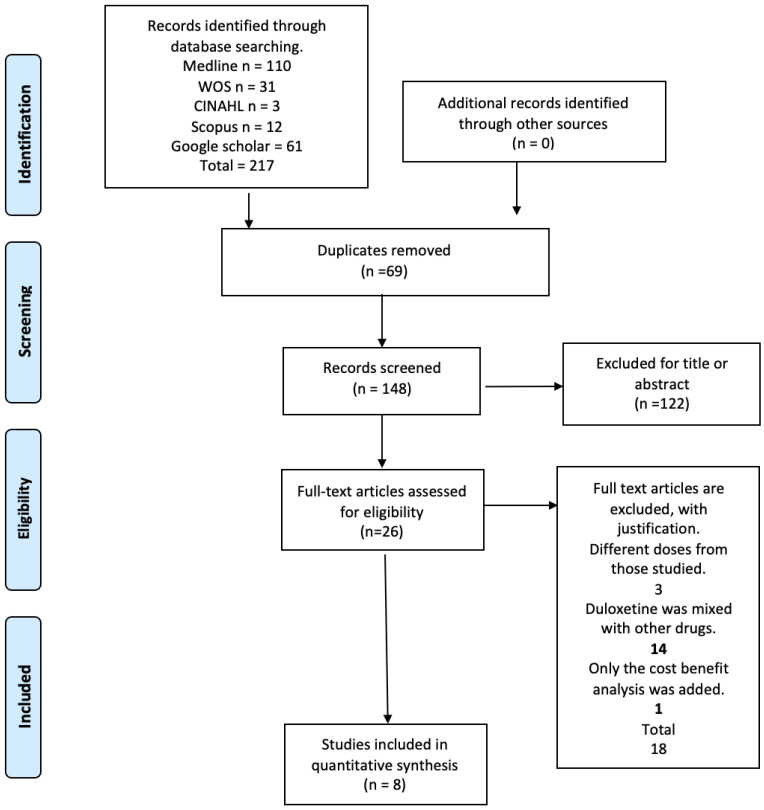
Flow diagram.

The electronic search retrieved a total of 217 articles, of which 26 were potentially eligible for full-text review. The detailed steps of the article selection process for the systematic review are described in a flow diagram (Figure 1). Finally, the present review included a total of eight randomized controlled trials that met the eligibility criteria [23,24,25,26,27,28,29,30]. The excluded studies and the reasons for their exclusion are available in Table S1 of the Supplementary Materials.

### 3.2. Study Characteristics

A summary of the included studies is presented in Table 1. The eight eligible studies compared several doses of duloxetine with a placebo or another type of treatment (gabapentin in different doses or routine care) [23,24,25,26,27,28,29,30]. The publication dates of the studies were from 2005 to 2020. The overall population included 2013 patients (1272 in the duloxetine group and 741 in the other therapeutic modalities group). The mean ages were 59 (±2.7) and 59.7 (±1.8) years for the duloxetine and the other therapeutic modalities groups, respectively. The mean follow-up duration was 24.8 months (ranging from 8 to 52).

### 3.3. Risk of Bias Assessment in Individual Studies

**Figure 2:**
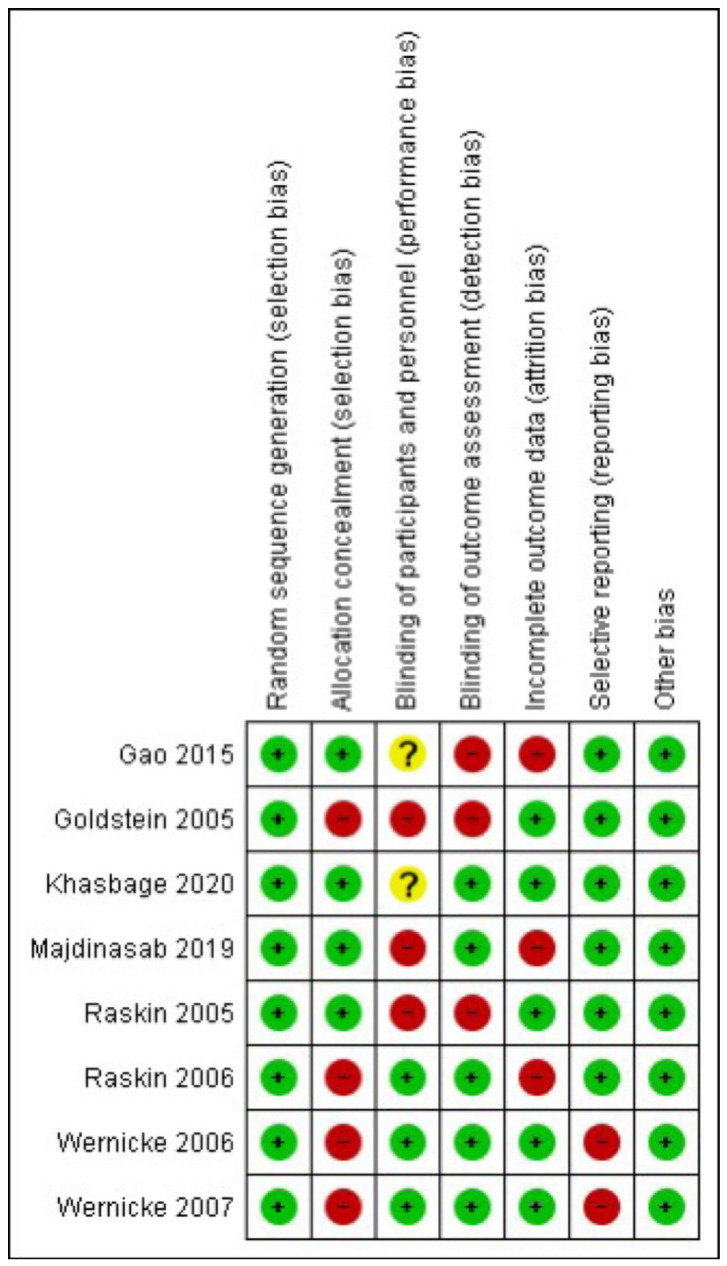
Risk of bias summary [23,24,25,26,27,28,29,30].

The RoB2 assessment is presented in Figure 2. In random sequence generation, 100% of the studies were classified as having “low risk” [23,24,25,26,27,28,29,30]; in allocation concealment, 50% were classified as having a “low risk” of bias [23,25,26,27], whereas 50% presented a “high risk” [24,28,29,30]. For blinding of participants and personnel, 37.5% of the trials received a “low risk” of bias rating [28,29,30], while 25% received “unclear” [23,25], whereas 37.5% presented a “high risk” [24,26,27]. For the blinding of outcome assessment, 62.5% of the trials were scored as “low risk” [25,26,28,29,30] and 37.5% as “high risk” [23,24,27]. For the incomplete outcome data, 62.5% received “low risk” [24,25,27,29,30] and 37.5% as “high risk” [23,26,28]. Finally, for the selection of the reported results, 75% of the trials were scored as “low risk” [23,24,25,26,27,28], and 25% were scored as having a “high” risk of bias [29,30].

### 3.4. Synthesis of Results

#### 3.4.1. 24-Hour Average Pain Severity Score

**Figure 3:**
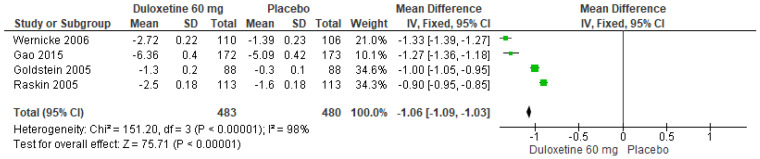
Forest plot of the effect of a 60 mg/d dose of duloxetine compared with a placebo on 24 h Average Pain Severity Score [23,24,27,29].

When a 60 mg/d dose of duloxetine was compared with a placebo, the four studies evaluated showed significant differences (SMD = −1.06; CI = −1.09 to −1.03; *p* < 0.00001; Figure 3) [23,24,27,29]. The direction of effect was consistent, and two of the CIs overlapped. Considerable statistical heterogeneity was observed (I^2^ = 98% and *p* < 0.00001). For this comparison, the funnel diagram graph showed an asymmetry, which indicates publication bias or factors that influence the results (Figure S1). The overall certainty of this evidence, based on the GRADE rating, was rated as very low quality of evidence (Table S3).

**Figure 4:**
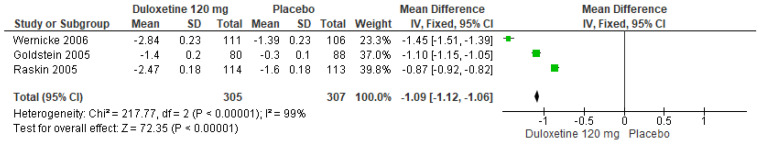
Forest plot of the effect of a 120 mg/d dose of duloxetine compared to a placebo on 24 h Average Pain Severity Score [24,27,29].

Similarly, for a 120 mg/d dose of duloxetine compared to a placebo, three studies expressed significant differences (SMD = −1.09; CI = −1.12 to −1.06; *p* < 0.00001; Figure 4) [24,27,29]. The direction of effect was equal in spite of the CIs not overlapping. Considerable statistical heterogeneity was observed (I^2^ = 99% and *p* < 0.00001). For this comparison, the funnel diagram graph showed an asymmetry, which indicates publication bias or factors that influence the results (Figure S2). The overall certainty of this evidence, based on the GRADE rating, was rated as very low quality of evidence (Table S3).

**Figure 5:**
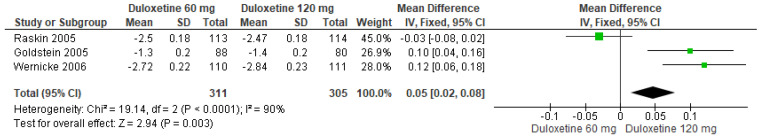
Forest plot of the effect of both doses of duloxetine (60 mg/d and 120 mg/d) compared with each other on 24 h Average Pain Severity Score [24,27,29].

Finally, when both doses of duloxetine (60 mg/d and 120 mg/d) were compared with each other, three studies showed significant differences (SMD = 0.05; CI = 0.02 to 0.08; *p* = 0.003; Figure 5) [24,27,29]. Two studies had the same direction of effect, and their CIs overlapped. Substantial statistical heterogeneity was observed (I^2^ = 90% and *p* < 0.0001). For this comparison, the funnel diagram graph showed an asymmetry, which indicates publication bias or factors that influence the results (Figure S3). The overall certainty of this evidence, based on the GRADE rating, was rated as low quality of evidence (Table S3).

#### 3.4.2. BPI Severity

**Figure 6:**
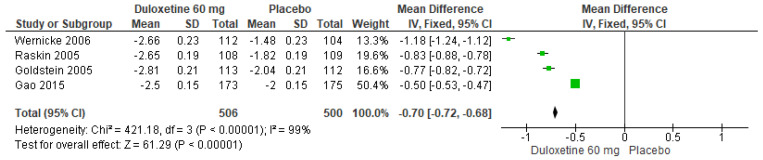
Forest plot of the effect of a 60 mg/d dose of duloxetine compared with a placebo on BPI Severity [23,24,27,29].

When 60 mg/d of duloxetine was compared with a placebo, four studies revealed significant differences (SMD = −0.70; CI = −0.72 to −0.68; *p* < 0.00001; Figure 6) [23,24,27,29]. The direction of effect from all studies was equal even though the CIs did not overlap. Considerable statistical heterogeneity was observed (I^2^ = 99% and *p* < 0.00001). For this comparison, the funnel diagram graph showed an asymmetry, which indicates publication bias or factors that influence the results (Figure S4). The overall certainty of this evidence, based on the GRADE rating, was rated as low quality of evidence (Table S3).

**Figure 7:**
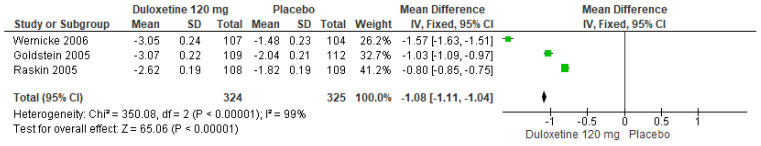
Forest plot of the effect of a 120 mg/d dose of duloxetine compared with a placebo on BPI Severity [24,27,29].

In a similar manner, for 120 mg/d of duloxetine compared to a placebo, three studies evaluated manifested significant differences (SMD = −1.08; CI = −1.11 to −1.04; *p* < 0.00001; Figure 7) [24,27,29]. There was a consistent direction of effect despite the fact that the CIs did not overlap. Considerable statistical heterogeneity was observed (I^2^ = 99% and *p* < 0.00001). For this comparison, the funnel diagram graph showed an asymmetry, which indicates publication bias or factors that influence the results (Figure S5). The overall certainty of this evidence, based on the GRADE rating, was rated as low quality of evidence (Table S3).

**Figure 8:**
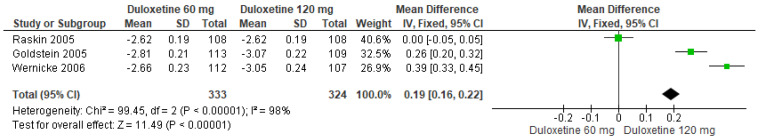
Forest plot of the effect of both doses of duloxetine (60 mg/d and 120 mg/d) compared with each other on BPI Severity [24,27,29].

Finally, when both doses of duloxetine (60 mg/d and 120 mg/d) were compared with each other, three studies evaluated showed significant differences (SMD = 0.19; CI = 0.16 to 0.22; *p* < 0.00001; Figure 8) [24,27,29]. Two studies had the same direction of effect with overlapping CIs, while one study had no tendency for any dose. Considerable statistical heterogeneity was observed (I^2^ = 98% and *p* < 0.0001). For this comparison, the funnel diagram graph showed an asymmetry, which indicates publication bias or factors that influence the results (Figure S6). The overall certainty of this evidence, based on the GRADE rating, was rated as very low quality of evidence (Table S3).

#### 3.4.3. BPI Interference

**Figure 9:**
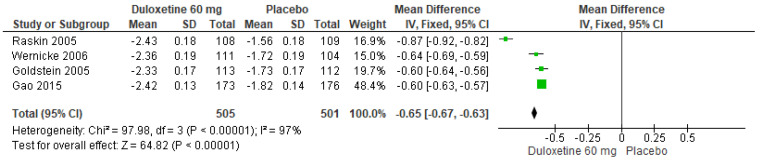
Forest plot of the effect of a 60 mg/d dose of duloxetine compared with a placebo on BPI Interference [23,24,27,29].

When 60 mg/d of duloxetine was compared with a placebo, four studies evaluated showed significant differences (SMD = −0.65; CI = −0.67 to −0.63; *p* < 0.00001; Figure 9) [23,24,27,29]. There was an equal direction of effect, and the CIs overlapped. Considerable statistical heterogeneity was observed (I^2^ = 97% and *p* < 0.00001). For this comparison, the funnel diagram graph showed an asymmetry, which indicates publication bias or factors that influence the results (Figure S7). The overall certainty of this evidence, based on the GRADE rating, was rated as very low quality of evidence (Table S3).

**Figure 10:**
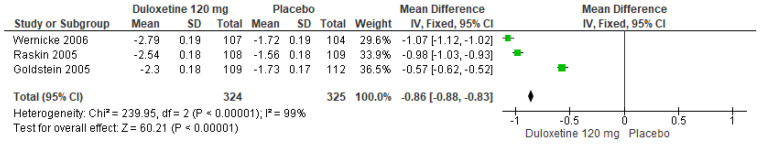
Forest plot of the effect of a 120 mg/d dose of duloxetine compared with a placebo on BPI Interference [24,27,29].

Likewise, for 120 mg/d of duloxetine compared to a placebo, three studies evaluated showed significant differences (SMD = −0.86; CI = −0.88 to −0.83; *p* < 0.00001; Figure 10) [24,27,29]. The direction of effect was consistent, and two studies had CIs that overlapped. Considerable statistical heterogeneity was observed (I^2^ = 99% and *p* < 0.00001). For this comparison, the funnel diagram graph showed an asymmetry, which indicates publication bias or factors that influence the results (Figure S8). The overall certainty of this evidence, based on the GRADE rating, was rated as low quality of evidence (Table S3).

**Figure 11:**
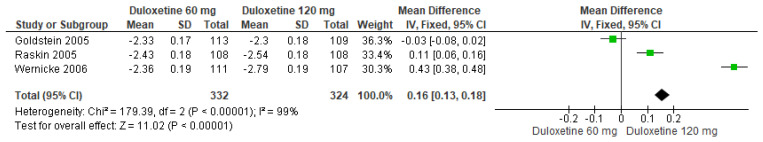
Forest plot of the effect of both doses of duloxetine (60 mg/d and 120 mg/d) compared with each other on BPI Interference [24,27,29].

Finally, when both doses of duloxetine (60 mg/d and 120 mg/d) were compared with each other, three studies expressed significant differences (SMD = 0.16; CI = 0.13 to 0.18; *p* < 0.00001; Figure 11) [24,27,29]. The direction of effect was not consistent in all studies, and the CIs did not overlap. Considerable statistical heterogeneity was observed (I^2^ = 99% and *p* < 0.0001). For this comparison, the funnel diagram graph showed an asymmetry, which indicates publication bias or factors that influence the results (Figure S9). The overall certainty of this evidence, based on the GRADE rating, was rated as very low quality of evidence (Table S3).

#### 3.4.4. CGI Severity

**Figure 12:**
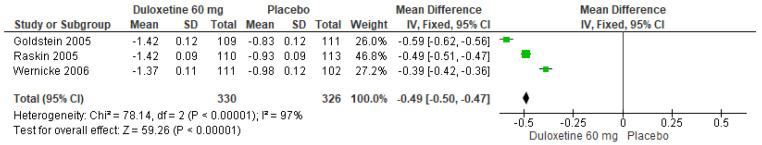
Forest plot of the effect of a 60 mg/d dose of duloxetine compared with a placebo on CGI Severity [24,27,29].

When 60 mg/d of duloxetine was compared with a placebo, three studies evaluated showed significant differences (SMD = −0.49; CI = −0.50 to −0.47; *p* < 0.00001; Figure 12) [24,27,29]. The direction of effect was consistent in spite of the CIs not overlapping. Considerable statistical heterogeneity was observed (I^2^ = 97% and *p* < 0.00001). For this comparison, the funnel diagram graph showed an asymmetry, which indicates publication bias or factors that influence the results (Figure S10). The overall certainty of this evidence, based on the GRADE rating, was rated as very low quality of evidence (Table S3).

**Figure 13:**
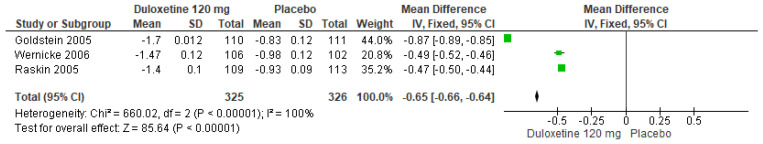
Forest plot of the effect of a 120 mg/d dose of duloxetine compared with a placebo on CGI Severity [24,27,29].

Similarly, for 120 mg/d of duloxetine compared to a placebo, three studies evaluated expressed significant differences (SMD = −0.65; CI = −0.66 to −0.64; *p* < 0.00001; Figure 13) [24,27,29]. There was an equal direction of effect, and two studies had overlapping CIs. Considerable statistical heterogeneity was observed (I^2^ = 100% and *p* < 0.00001). For this comparison, the funnel diagram graph showed an asymmetry, which indicates publication bias or factors that influence the results (Figure S11). The overall certainty of this evidence, based on the GRADE rating, was rated as low quality of evidence (Table S3).

**Figure 14:**
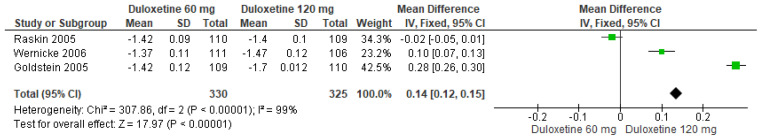
Forest plot of the effect of both doses of duloxetine (60 mg/d and 120 mg/d) compared with each other on CGI Severity [24,27,29].

Finally, when both doses of duloxetine (60 mg/d and 120 mg/d) were compared with each other, three studies evaluated manifested significant differences (SMD = 0.14; CI = 0.12 to 0.15; *p* < 0.00001; Figure 14) [24,27,29]. The direction of effect was equal for two studies only, and the CIs did not overlap. Considerable statistical heterogeneity was observed (I^2^ = 99% and *p* < 0.0001). For this comparison, the funnel diagram graph showed an asymmetry, which indicates publication bias or factors that influence the results (Figure S12). The overall certainty of this evidence, based on the GRADE rating, was rated as very low quality of evidence (Table S3).

#### 3.4.5. PGI Improvement 

**Figure 15:**
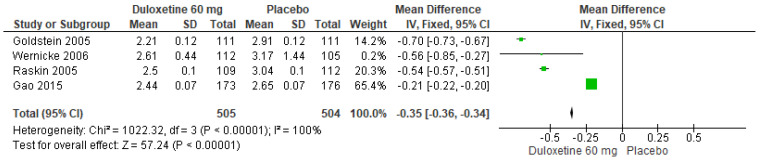
Forest plot of the effect of a 60 mg/d dose of duloxetine compared with a placebo on PGI Improvement [23,24,27,29].

When 60 mg/d of duloxetine was compared with a placebo, four studies evaluated showed significant differences (SMD = −0.35; CI = −0.36 to −0.34; *p* < 0.00001; Figure 15) [23,24,27,29]. There was a consistent direction of effect, and the CIs overlapped. Considerable statistical heterogeneity was observed (I^2^ = 100% and *p* < 0.00001). For this comparison, the funnel diagram graph showed an asymmetry, which indicates publication bias or factors that influence the results (Figure S13). The overall certainty of this evidence, based on the GRADE rating, was rated as low quality of evidence (Table S3).

**Figure 16:**
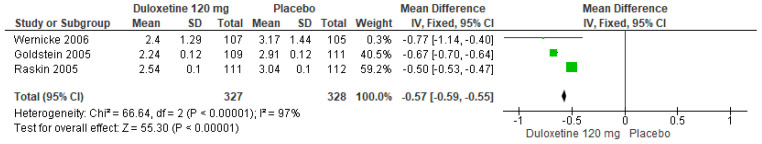
Forest plot of the effect of a 120 mg/d dose of duloxetine compared with a placebo on PGI Improvement [24,27,29].

In a similar manner, for 120 mg/d of duloxetine compared to a placebo, three studies evaluated expressed significant differences (SMD = −0.57; CI = −0.59 to −0.55; *p* < 0.00001; Figure 16) [24,27,29]. The direction of effect was consistent, and the CIs overlapped. Considerable statistical heterogeneity was observed (I^2^ = 97% and *p* < 0.00001). For this comparison, the funnel diagram graph showed an asymmetry, which indicates publication bias or factors that influence the results (Figure S14). The overall certainty of this evidence, based on the GRADE rating, was rated as low quality of evidence (Table S3).

**Figure 17:**
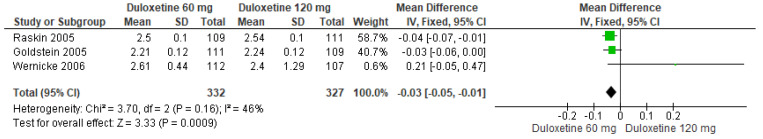
Forest plot of the effect of both doses of duloxetine (60 mg/d and 120 mg/d) compared with each other on PGI Improvement [24,27,29].

Finally, when both doses of duloxetine (60 mg/d and 120 mg/d) were compared with each other, three studies showed significant differences (SMD = −0.03; CI = −0.05 to −0.01; *p* = 0.0009; Figure 17) [24,27,29]. The direction of effect was not consistent, but the CIs overlapped. Moderate statistical heterogeneity was observed (I^2^ = 46% and *p* = 0.16). For this comparison, the funnel plot graph showed symmetry, indicating the absence of publication bias or factors influencing the results (Figure S15). The overall certainty of this evidence, based on the GRADE rating, was rated as low quality of evidence (Table S3).

#### 3.4.6. SF-MPQ Total Score

**Figure 18:**
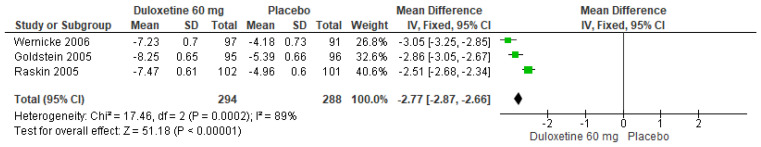
Forest plot of the effect of a 60 mg/d dose of duloxetine compared with a placebo on SF-MPQ Total Score [24,27,29].

When 60 mg/d of duloxetine was compared with a placebo, three studies evaluated manifested significant differences (SMD = −2.77; CI = −2.87 to −2.66; *p* < 0.00001; Figure 18) [24,27,29]. The direction of effect from all studies was equal, and the CIs overlapped. Considerable statistical heterogeneity was observed (I^2^ = 89% and *p* = 0.0002). For this comparison, the funnel diagram graph showed an asymmetry, which indicates publication bias or factors that influence the results (Figure S16). The overall certainty of this evidence, based on the GRADE rating, was rated as low quality of evidence (Table S3).

**Figure 19:**
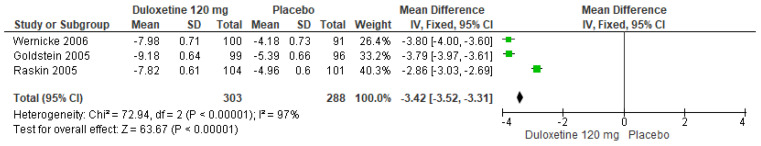
Forest plot of the effect of a 120 mg/d dose of duloxetine compared with a placebo on SF-MPQ Total Score [24,27,29].

Likewise, for 120 mg/d of duloxetine compared to a placebo, three studies expressed significant differences (SMD = −3.42; CI = −3.52 to −3.31; *p* < 0.00001; Figure 19) [24,27,29]. The direction of effect was consistent, and two studies had overlapping CIs. Considerable statistical heterogeneity was observed (I^2^ = 97% and *p* < 0.00001). For this comparison, the funnel diagram graph showed an asymmetry, which indicates publication bias or factors that influence the results (Figure S17). The overall certainty of this evidence, based on the GRADE rating, was rated as low quality of evidence (Table S3).

**Figure 20:**
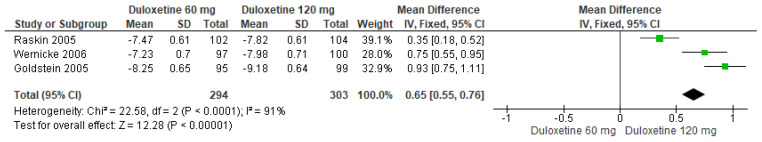
Forest plot of the effect of both doses of duloxetine (60 mg/d and 120 mg/d) compared with each other on SF-MPQ Total Score [24,27,29].

Finally, when both doses of duloxetine (60 mg/d and 120 mg/d) were compared with each other, three studies manifested significant differences (SMD = 0.65; CI = 0.55 to 0.76; *p* < 0.0001; Figure 20) [24,27,29]. The direction of effect from all studies was consistent, and the CIs overlapped. Considerable statistical heterogeneity was observed (I^2^ = 91% and *p* < 0.0001). For this comparison, the funnel diagram graph showed an asymmetry, which indicates publication bias or factors that influence the results (Figure S18). The overall certainty of this evidence, based on the GRADE rating, was rated as low quality of evidence (Table S3).

#### 3.4.7. Euro Quality of Life Questionnaire

**Figure 21:**
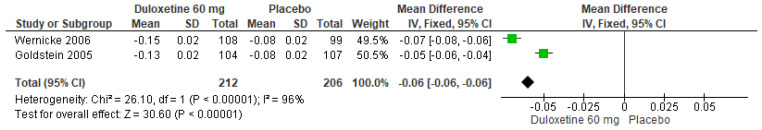
Forest plot of the effect of a 60 mg/d dose of duloxetine compared with a placebo on the Euro Quality of Life Questionnaire [24,29].

When 60 mg/d of duloxetine was compared with a placebo, two studies expressed significant differences (SMD = −0.06; CI = −0.06 to −0.06; *p* < 0.00001; Figure 21) [24,29]. There was an equal direction of effect, but the CIs did not overlap. Considerable statistical heterogeneity was observed (I^2^ = 96% and *p* < 0.00001). For this comparison, the funnel plot graph showed symmetry, indicating the absence of publication bias or factors influencing the results (Figure S19). The overall certainty of this evidence, based on the GRADE rating, was rated as very low quality of evidence (Table S3).

**Figure 22:**
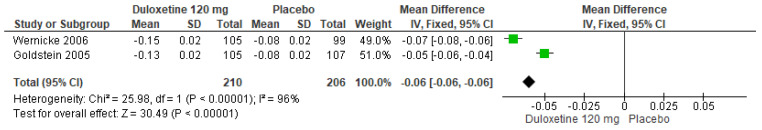
Forest plot of the effect of a 120 mg/d dose of duloxetine compared with a placebo on the Euro Quality of Life Questionnaire [24,29].

In a similar manner, for 120 mg/d of duloxetine compared to a placebo, two studies showed significant differences (SMD = −0.06; CI = −0.06 to −0.06; *p* < 0.00001; Figure 22) [24,29]. The direction of effect was equal, but the CIs did not overlap. Considerable statistical heterogeneity was observed (I^2^ = 96% and *p* < 0.00001). For this comparison, the funnel plot graph showed symmetry, indicating the absence of publication bias or factors influencing the results (Figure S20). The overall certainty of this evidence, based on the GRADE rating, was rated as very low quality of evidence (Table S3).

**Figure 23:**
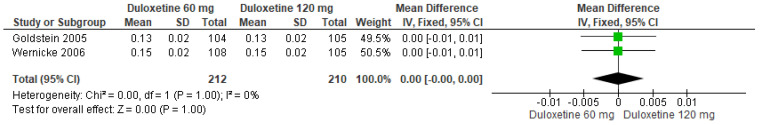
Forest plot of the effect of both doses of duloxetine (60 mg/d and 120 mg/d) compared with each other on the Euro Quality of Life Questionnaire [24,29].

Likewise, when both doses of duloxetine (60 mg/d and 120 mg/d) were compared with each other, two studies showed no significant differences (SMD = 0.00; CI = −0.00 to 0.00; *p* = 1.00; Figure 23) [24,29]. The direction of effect was null, and the statistical heterogeneity observed may not be important (I^2^ = 0% and *p* = 1.00). For this comparison, the funnel plot graph showed symmetry, indicating the absence of publication bias or factors influencing the results (Figure S21). The overall certainty of this evidence, based on the GRADE rating, was rated as very low quality of evidence (Table S3).

**Figure 24:**
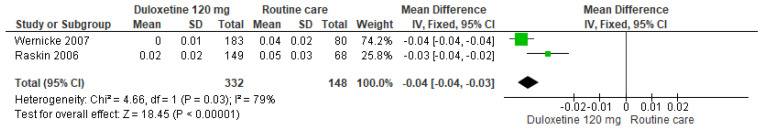
Forest plot of the effect of a 120 mg/d dose of duloxetine compared to routine care on the Euro Quality of Life Questionnaire [28,30].

Finally, for 120 mg/d of duloxetine compared to routine care, two studies showed significant differences (SMD = −0.04; CI = −0.03 to −0.04; *p* < 0.00001; Figure 24) [28,30]. The direction of effect from all studies was consistent, but the CIs did not overlap. Substantial statistical heterogeneity was observed (I^2^ = 79% and *p* = 0.03). For this comparison, the funnel plot graph showed symmetry, indicating the absence of publication bias or factors influencing the results (Figure S22). The overall certainty of this evidence, based on the GRADE rating, was rated as very low quality of evidence (Table S3).

#### 3.4.8. SF-36 Survey Bodily Pain

**Figure 25:**
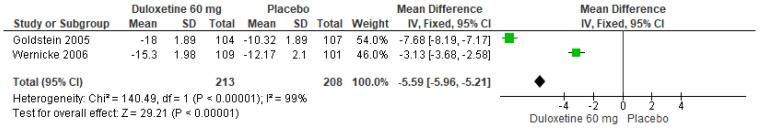
Forest plot of the effect of a 60 mg/d dose of duloxetine compared with a placebo on SF-36 Survey Bodily Pain [24,29].

When 60 mg/d of duloxetine was compared with a placebo, two studies expressed significant differences (SMD = −5.59; CI = −5.96 to −5.21; *p* < 0.00001; Figure 25) [24,29]. There was an equal direction of effect even though the CIs did not overlap. Considerate statistical heterogeneity was observed (I^2^ = 99% and *p* < 0.00001). For this comparison, the funnel diagram graph showed an asymmetry, which indicates publication bias or factors that influence the results (Figure S23). The overall certainty of this evidence, based on the GRADE rating, was rated as low quality of evidence (Table S3).

**Figure 26:**
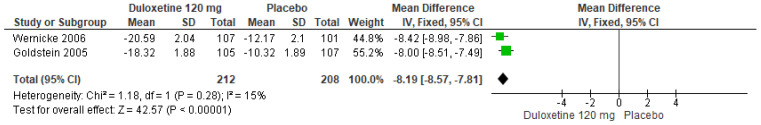
Forest plot of the effect of a 120 mg/d dose of duloxetine compared with a placebo on SF-36 Survey Bodily Pain [24,29].

Similarly, for 120 mg/d of duloxetine compared to a placebo, two studies expressed significant differences (SMD = −8.19; CI = −8.57 to −7.81; *p* < 0.00001; Figure 26) [24,29]. There was a consistent direction of effect from all studies, and the CIs overlapped. The statistical heterogeneity observed may not be important (I^2^ = 15% and *p* = 0.28). For this comparison, the funnel plot graph showed symmetry, indicating the absence of publication bias or factors influencing the results (Figure S24). The overall certainty of this evidence, based on the GRADE rating, was rated as very low quality of evidence (Table S3).

**Figure 27:**
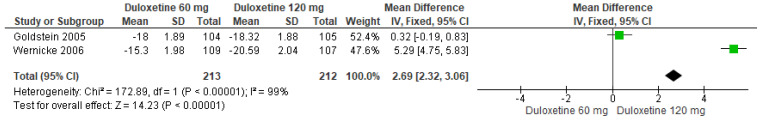
Forest plot of the effect of both doses of duloxetine (60 mg/d and 120 mg/d) compared with each other on SF-36 Survey Bodily Pain [24,29].

Likewise, when both doses of duloxetine (60 mg/d and 120 mg/d) were compared with each other, two studies showed significant differences (SMD = 2.69; CI = 2.32 to 3.06; *p* < 0.0001; Figure 27) [24,29]. The direction of effect was consistent, but the CIs did not overlap. Considerable statistical heterogeneity was observed (I^2^ = 99% and *p* < 0.0001). For this comparison, the funnel diagram graph showed an asymmetry, which indicates publication bias or factors that influence the results (Figure S25). The overall certainty of this evidence, based on the GRADE rating, was rated as low quality of evidence (Table S3).

**Figure 28:**
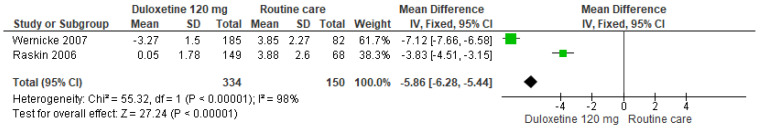
Forest plot of the effect of a 120 mg/d dose of duloxetine compared to routine care on SF-36 Survey Bodily Pain [28,30].

Finally, for 120 mg/d of duloxetine compared to routine care, two studies showed significant differences (SMD = −5.86; CI = −6.28 to −5.44; *p* < 0.00001; Figure 28) [28,30]. The direction of effect was equal in spite of the CIs not overlapping. Considerable statistical heterogeneity was observed (I^2^ = 98% and *p* < 0.00001). For this comparison, the funnel diagram graph showed an asymmetry, which indicates publication bias or factors that influence the results (Figure S26). The overall certainty of this evidence, based on the GRADE rating, was rated as low quality of evidence (Table S3).

#### 3.4.9. VAS

**Figure 29:**
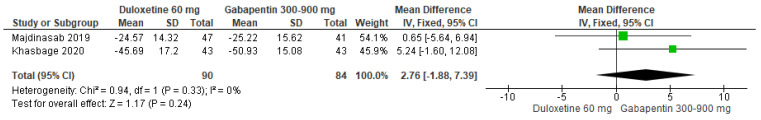
Forest plot of the effect of a 60 mg/d dose of duloxetine compared with doses of 300 to 900 mg/d of gabapentin on VAS [25,26].

When a 60 mg/d dose of duloxetine was compared with doses of 300 to 900 mg/d of gabapentin, two studies expressed no significant differences (SMD = 2.76; CI = −1.88 to 7.39; *p* = 0.24; Figure 29) [25,26]. The direction of effect was consistent and in favor of gabapentin with the CIs overlapped. The statistical heterogeneity observed may not be important (I^2^ = 0% and *p* = 0.33). For this comparison, the funnel plot graph showed symmetry, indicating the absence of publication bias or factors influencing the results (Figure S27). The overall certainty of this evidence, based on the GRADE rating, was rated as very low quality of evidence (Table S3).

## 4. Discussion

This systematic review aimed to learn about the efficacy of duloxetine for the management of NP associated with DM. The main results demonstrate that duloxetine, in most interventions, was significantly more beneficial compared to a placebo; however, they should be taken with caution in relation to what is discussed below.

After performing the bibliographic search, nine systematic review or meta-analysis studies were found that studied the use of duloxetine in the treatment of NP. These studies, in their title or abstract, compared duloxetine with some other therapeutic modality. Of these studies, only one did not directly evaluate NP but evaluated plasma glucose levels in patients with DM [31], which makes this work completely different from the objective of our review, so that the perspective we offer is more focused on clinical practice.

Secondly, there were two studies comparing duloxetine with gabapentin for the treatment of DNP. The study by Jiang [32] found a slight but greater efficacy of duloxetine, which was supported by statistically significant differences compared to gabapentin. In contrast, the present study also uses a placebo as a control group. In addition, our analysis favored gabapentin as the most effective therapy, with a greater effect on pain reduction, so further studies are needed to be able to have a more accurate correlation of this comparison. The study by Quilici [33] combined duloxetine, pregabalin, and gabapentin, and the results showed that there are no significant differences between the use of gabapentin or duloxetine for the treatment of DNP, but there is no consensus regarding pregabalin. The results of the latter were controversial, as some results of certain pain scales were favorable to pregabalin; other scales showed no significant differences in their results, highlighting the complexity of the comparison between the different therapeutic modalities. However, when comparing duloxetine, gabapentin, and pregabalin with the placebo group, they did show a trend in favor of decreasing NP in patients with DM. Although these results are controversial, our results cannot be compared with them since none of the included studies compared duloxetine with the mixture of pregabalin and gabapentin, thereby highlighting the importance of considering factors such as the treatment modalities in clinical decision making.

To conclude the comparison of the present study with previous ones, six studies compared duloxetine with a placebo [34,35,36,37,38,39], which conclusively confirmed that duloxetine can significantly improve functional status and quality of life in patients with DPN [34,36,37], as well as the safety and tolerability of this drug in DM patients with chronic NP [35,38,39]. In contrast to previous studies, the present review included recent studies in addition to those addressed by older reviews. It also considers a wide range of pain, symptom improvement, and quality of life scales, which allow a comprehensive view of patients with NP due to DM who are treated with duloxetine.

In relation to the results found in this review, all studies comparing the use of duloxetine versus a placebo revealed a clear benefit in favor of duloxetine, consistent in all the pain scales analyzed. In each of these scales, the difference in favor of duloxetine was statistically significant, strongly confirming the efficacy of duloxetine in reducing NP in patients with DM. These findings support the incorporation of duloxetine as an effective therapeutic option in the management of DPN.

Additionally, clinical trials comparing different doses of duloxetine with each other for the treatment of DNP, corresponding to 60 and 120 mg/d, were analyzed. Most of the results showed a tendency in favor of the 120 mg dose compared to the lower dose in different pain scales. However, there were cases where the differences were not statistically significant. There was no trend toward any dose, and the pain scales evaluated showed a tendency to favor the lower dose. These variations can be explained by the duration of treatment, frequency of administration, other comorbidities, and severity of DNP at baseline, among other factors.

Other studies compared duloxetine with routine care for the treatment of NP. It is important to note that the latter included a broad spectrum of therapies that the investigator and patient agreed were optimal for symptom improvement. Although the studies report no significant differences between the duloxetine and routine care groups, the set of data analyzed by the forest plot showed a tendency toward duloxetine with statistically significant differences. This discrepancy between the results of the individual studies and the meta-analysis can be explained by the heterogeneity of the interventions in the routine care group, or even the treatment effects may have been overestimated, underscoring the importance of interpreting these results with caution before making definitive conclusions [37,38,39].

Finally, duloxetine was compared with gabapentin, the latter showing the greatest benefits in terms of the reduction in NP associated with DM on the Visual Analog Scale. Despite this, the difference was not significant between the treatments, which may be due to variability in individual patient response to each drug, so it is necessary to consider individual patient characteristics in choosing the most appropriate therapy in the management of DNP.

From the pharmacokinetic point of view, duloxetine is a powerful antidepressant, which reaches peak plasma concentrations between 47 ng/mL and 110 ng/mL, is metabolized via cytochrome P450, and is eliminated via the urinary tract [40]. In addition to being used in the treatment of DPN, it is effective in the treatment of depression, generalized anxiety, and fibromyalgia, among others [41]. Its extensive study and acceptance in clinical practice have led to a deep understanding of its effect on cellular physiology. However, it is important to recognize there could be other parameters that influence its therapeutic effect, such as the emotional factor, concomitant treatments, other pathologies with the same type of metabolization, and other particular aspects of the patient, which may play a crucial role in the desired response of the treatment for pain with duloxetine.

Therefore, despite the extensive use of duloxetine in clinical practice and the results presented in this review, further studies are needed to fully investigate its efficacy and safety. Furthermore, it is essential to evaluate its clinical effect in a particular way in each patient, in addition to the statistical effects. This implies considering both the quantitative aspects offered by this meta-analysis and the qualitative aspects of each patient’s response to treatment, which influence their perception of pain and the efficacy of the treatment, in order to improve the quality of life of affected patients.

## 5. Limitations

This review was limited by the publication and authorship bias of the included studies. Firstly, studies with different results that were in the non-indexed literature in the selected databases may have been excluded. Secondly, there may have been limitations in the sensitivity and specificity of the searches. Finally, the authors personally selected articles. All of this increases the probability of excluding potential cases from countries outside of Asia and North America that have not been reported in the scientific community. In addition, the primary studies do not clearly and homogeneously specify the concurrent pathology of the patients, the duration of the disease, the degree of glycemia and glycated hemoglobin, and other factors related to diabetes mellitus.

## 6. Conclusions

In summary, duloxetine is statistically and clinically an effective therapeutic option for the management of NP associated with DM. However, new studies, including quantitative and qualitative parameters to assess pain reduction and improvement in patients’ quality of life, are needed to better support these results.

## Figures and Tables

**Table 1:** Summary of the characteristics of the studies included in the meta-analysis. DM, diabetes mellitus; NP, neuropathic pain.

Author, Year, and Region	Type of Study and N	Incidence and Characteristics	Statistical Values (Mean and Standard Deviation)		Baseline Sex, N (%)	Doses
Gao et al., 2015 [23] China	Randomized Clinical Trial Total (initial): N = 405 Total (final): N = 349	DM patients with diabetic NP. Daily pain had to be present for at least 6 months.	24 h Average Pain Severity - Placebo Baseline: 10.5 (7.3) Change: −5.09 (0.42) - Duloxetine Baseline: 11.2 (7.6) Change: −6.36 (0.40) BPI Severity - Placebo Baseline: 5.9 (1.6) Change: −2.00 (0.15) - Duloxetine Baseline: 6.0 (1.7) Change: −2.50 (0.15)	BPI Interference - Placebo Baseline: 4.1 (2.3) Change: −1.82 (0.14) - Duloxetine Baseline: 4.4 (2.3) Change: −2.42 (0.13) PGI Improvement - Placebo Change: 2.65 (0.07) - Duloxetine Change: 2.44 (0.07)	Placebo: M = 91 (45.0) F = 111 (55.0) Duloxetine: M = 91 (44.8) F = 112 (55.2)	Duloxetine Group: 11 weeks of 60 mg/d after a week of 30 mg/d. Placebo Group: Placebo once daily. 12 weeks of administration.
Goldstein et al., 2005 [24] Multicenter	Randomized Clinical Trial Total (initial): N = 457 Total (final): N = 344	DM patients with diabetic NP. Daily pain had to be present for at least 6 months.	24 h Pain Severity Score - Placebo Baseline: 5.8 (1.5) Change: −0.3 (0.1) - Duloxetine 60 mg/d Baseline: 6.0 (1.7) Change: −1.3 (0.2) - Duloxetine 120 mg/d Baseline: 5.9 (1.4) Change: −1.4 (0.2) BPI Severity - Placebo Change: −2.04 (0.21) - Duloxetine 60 mg/d Change: −2.81 (0.21) - Duloxetine 120 mg/d Change: −3.07 (0.22) BPI Interference - Placebo Change: −1.73 (0.17) - Duloxetine 60 mg/d Change: −2.33 (0.17) - Duloxetine 120 mg/d Change: −2.30 (0.18) CGI Severity - Placebo Baseline: 4.4 (0.9) Change: −0.83 (0.12) - Duloxetine 60 mg/d Baseline: 4.3 (1.0) Change: −1.42 (0.12) - Duloxetine 120 mg/d Baseline: 4.4 (0.9) Change: −1.70 (0.012)	PGI Improvement - Placebo Change: 2.91 (0.12) - Duloxetine 60 mg/d Change: 2.21 (0.12) - Duloxetine 120 mg/d Change: 2.24 (0.12) SF-MPQ Total Score - Placebo Change: −5.39 (0.66) - Duloxetine 20 mg/d Change: −7.23 (0.67) - Duloxetine 60 mg/d Change: −8.25 (0.65) - Duloxetine 120 mg/d Change: −9.18 (0.64) Euro Quality of Life Questionnaire - Placebo Change: 0.08 (0.02) - Duloxetine 60 mg/d Change: 0.13 (0.02) - Duloxetine 120 mg/d Change: 0.13 (0.02) SF-36 Survey Bodily Pain - Placebo Change: 10.32 (1.89) - Duloxetine 60 mg/d Change: 18.00 (1.89) - Duloxetine 120 mg/d Change: 18.32 (1.88)	Placebo: M = 59 (51.3) F = 56 (48.7) 60 mg: M = 79 (69.3) F = 35 (30.7) 120 mg: M = 68 (60.2) F = 45 (39.8)	Duloxetine Groups: - 60 mg/d. - 120 mg/d. Placebo Group: Placebo once daily. 12 weeks of administration.
Khasbage et al., 2020 [25] India	Randomized Clinical Trial Total (initial): N = 86 Total (final): N = 81	Type II DM patients with diabetic NP. Daily pain had to be present for at least 1 month.	VAS - Duloxetine Baseline: 72.44 (8.47) Final: 26.86 (16.69) Change: 45.69 (17.20) - Gabapentin Baseline: 73.37 (10.56) Final: 22.442 (14.36) Change: 50.93 (15.08)		Duloxetine: M = 27 (62.8) F = 16 (37.2) Gabapentin: M = 25 (58.1) F = 18 (41.9)	Duloxetine Group: 60 mg/d at bedtime. Gabapentin Group: 300 mg/d at bedtime. 12 weeks of administration.
Majdinasab et al., 2019 [26] Iran	Randomized Clinical Trial Total (initial): N = 104 Total (final): N = 88	DM patients with diabetic NP. Daily pain had to be present between 1 month and 5 years.	VAS - Gabapentin Baseline: 64 (20.03) Week 8: 39.43 (14.32) - Duloxetine Baseline: 62 (21.18) Week 8: 36.78 (15.62)		Gabapentin: M = 21 (40.4) F = 31 (59.6) Duloxetine: M = 24 (46.2) F = 28 (53.8)	Duloxetine Group: 7 weeks of 60 mg/d at bedtime after a week of 30 mg/d. Gabapentin Group: 300–900 mg/d at bedtime 8 weeks of oral administration.
Raskin et al., 2005 [27] Canada	Randomized Clinical Trial Total (initial): N = 348 Total (final): N = 296	DM patients with bilateral peripheral neuropathy. Daily pain had to be present for at least 6 months.	24 h Pain Severity Score - Placebo Baseline: 5.5 (1.3) Change: −1.60 (0.18) - Duloxetine 60 mg QD Baseline: 5.5 (1.1) Change: −2.50 (0.18) - Duloxetine 60 mg BID Baseline: 5.7 (1.3) Change: −2.47 (0.18) BPI Severity - Placebo Change: −1.82 (0.19) - Duloxetine 60 mg QD Change: −2.65 (0.19) - Duloxetine 60 mg BID Change: −2.62 (0.19) BPI Interference - Placebo Change: −1.56 (0.18) - Duloxetine 60 mg QD Change: −2.43 (0.18) - Duloxetine 60 mg BID Change: −2.54 (0.18)	CGI Severity - Placebo Baseline: 4.5 (0.9) Change: −0.93 (0.09) - Duloxetine 60 mg QD Baseline: 4.6 (0.9) Change: −1.42 (0.09) - Duloxetine 60 mg BID Baseline: 4.5 (1.0) Change: −1.40 (0.10) PGI Improvement - Placebo Change: 3.04 (0.10) - Duloxetine 60 mg QD Change: 2.50 (0.10) - Duloxetine 60 mg BID Change: 2.54 (0.10) SF-MPQ Total Score - Placebo Change: −4.96 (0.60) - Duloxetine 60 mg QD Change: −7.47 (0.61) - Duloxetine 60 mg BID Change: −7.82 (0.61)	Placebo: M = 53 (45.7) F = 63 (54.3) Duloxetine 60 mg QD: M = 48 (41.4) F = 68 (58.6) Duloxetine 60 mg BID: M = 61 (52.6) F = 55 (47.4) Total: M = 162 (46.6) F = 186 (53.4)	Duloxetine Groups: - 60 mg/d. - 120 mg/d. Placebo Group: Placebo twice daily. 12 weeks of oral administration.
Raskin et al., 2006 [28] Multicenter (United States and Puerto Rico)	Randomized Clinical Trial Total (initial): N = 237 Total (final): N = 179	DM patients with diabetic NP. Daily pain had to be present for at least 6 months.	Euro Quality of Life Questionnaire - Duloxetine Change: −0.02 (0.02) - Routine care Change: −0.05 (0.03)	SF-36 Bodily Pain - Duloxetine Change: −0.05 (1.78) - Routine care Change: −3.88 (2.60)	Routine Care: M = 46 (60.5) F = 30 (39.5) Duloxetine M = 99 (61.5) F = 62 (38.5)	After a 13-week therapy with duloxetine and a placebo, patients were assigned as follows: Duloxetine Group: 120 mg/d. Routine Care Group: Therapies that the investigator and patients considered optimal, including gabapentin, amitriptyline, and venlafaxine.
Wernicke et al., 2006 [29] Multicenter	Randomized Clinical Trial Total (initial): N = 334 Total (final): N = 248	DM patients with diabetic peripheral NP. Daily pain had to be present for at least 6 months.	24 h Pain Severity Score - Placebo Baseline: 5.9 (1.4) Change: −1.39 (0.23) - Duloxetine 60 mg QD Baseline: 6.1 (1.6) Change: −2.72 (0.22) - Duloxetine 60 mg BID Baseline: 6.2 (1.5) Change: −2.84 (0.23) BPI Severity - Placebo Change: −1.48 (0.23) - Duloxetine 60 mg QD Change: −2.66 (0.23) - Duloxetine 60 mg BID Change: −3.05 (0.24) BPI Interference - Placebo Baseline: 4.2 (2.2) Change: −1.72 (0.19) - Duloxetine 60 mg QD Baseline: 4.7 (2.5) Change: −2.36 (0.19) - Duloxetine 60 mg BID Baseline: 5.0 (2.4) Change: −2.79 (0.19) CGI Severity - Placebo Baseline: 4.6 (0.8) Change: −0.98 (0.12) - Duloxetine 60 mg QD Baseline: 4.5 (0.9) Change: −1.37 (0.11) - Duloxetine 60 mg BID Baseline: 4.6 (0.8) Change: −1.47 (0.12)	PGI Improvement - Placebo Change: 3.17 (1.44) - Duloxetine 60 mg QD Change: 2.61 (0.44) - Duloxetine 60 mg BID Change: 2.40 (1.29) SF-MPQ Total Score - Placebo Baseline: 16.2 (7.5) Change: −4.18 (0.73) - Duloxetine 60 mg QD Baseline: 15.9 (7.7) Change: −7.23 (0.70) - Duloxetine 60 mg BID Baseline: 16.8 (6.7) Change: −7.98 (0.71) Euro Quality of Life Questionnaire - Placebo Change: 0.08 (0.02) - Duloxetine 60 mg QD Change: 0.15 (0.02) - Duloxetine 60 mg BID Change: 0.15 (0.02) SF-36 Bodily pain - Placebo Change: 12.17 (2.10) - Duloxetine 60 mg QD Change: 15.30 (1.98) - Duloxetine 60 mg BID Change: 20.59 (2.04)	Placebo M = 39 (36.1) F = 69 (63.9) Duloxetine 60 mg QD M = 40 (35.1) F = 74 (64.9) Duloxetine 60 mg BID M = 51 (45.5) F = 61 (54.5)	Duloxetine Group: - 11 weeks of 60 mg/d and a final week of 30 mg/d. - 11 weeks of 120 mg/d and a final week of 60 mg/d. Placebo Group: Placebo once daily. 12 weeks of administration.
Wernicke et al., 2007 [30] Multicenter (Canada, Croatia, Hungary, Poland, Germany, and the Russian Federation)	Randomized Clinical Trial Total (initial): N = 293 Total (final): N = 259	DM patients with bilateral peripheral neuropathy. Daily pain had to be present for at least 6 months.	Euro quality of life Questionnaire - Duloxetine Change: 0.00 (0.01) - Routine care Change: −0.04 (0.02)	SF-36 Bodily pain - Duloxetine Change: 3.27 (1.50) - Routine care Change: −3.85 (2.27)	Duloxetine: M = 88 (44.7) F = 109 (55.3) Routine care: M = 47 (49.0) F = 49 (51.0)	After a 13-week therapy with a placebo, patients were assigned as follows: Duloxetine Group: 120 mg/d after 3 days of 30 mg/d. Routine Care Group: Therapies that the investigator and patients considered optimal. 52 weeks of oral administration.

## Supplementary Materials

The following supporting information can be downloaded at https://www.mdpi.com/article/10.3390/ph17070856/s1, References [42,43,44,45,46,47,48,49,50,51,52,53,54,55,56,57,58,59] are cited in the supplementary materials. Figure S1. Funnel plot of the effect of 60 mg/d dose of duloxetine compared with placebo on 24-hour Average Pain Severity Score. Figure S2. Funnel plot of the effect of 120 mg/d dose of duloxetine compared to placebo on 24-hour Average Pain Severity Score. Figure S3. Funnel plot of the effect of both doses of duloxetine (60 mg/d and 120 mg/d) compared with each other on 24-hour Average Pain Severity Score. Figure S4. Funnel plot of the effect of 60 mg/d dose of duloxetine compared with placebo on BPI Severity. Figure S5. Funnel plot of the effect of 120 mg/d dose of duloxetine compared with placebo on BPI Severity. Figure S6. Funnel plot of the effect of both doses of duloxetine (60 mg/d and 120 mg/d) compared with each other on BPI Severity. Figure S7. Funnel plot of the effect of 60 mg/d dose of duloxetine compared with placebo on BPI Interference. Figure S8. Funnel plot of the effect of 120 mg/d dose of duloxetine compared with placebo on BPI Interference. Figure S9. Funnel plot of the effect of both doses of duloxetine (60 mg/d and 120 mg/d) compared with each other on BPI Interference. Figure S10. Funnel plot of the effect of 60 mg/d dose of duloxetine compared with placebo on CGI Severity. Figure S11. Funnel plot of the effect of 120 mg/d dose of duloxetine compared with placebo on CGI Severity. Figure S12. Funnel plot of the effect of both doses of duloxetine (60 mg/d and 120 mg/d) compared with each other on CGI Severity. Figure S13. Funnel plot of the effect of 60 mg/d dose of duloxetine compared with placebo on PGI Improvement. Figure S14. Funnel plot of the effect of 120 mg/d dose of duloxetine compared with placebo on PGI Improvement. Figure S15. Funnel plot of the effect of both doses of duloxetine (60 mg/d and 120 mg/d) compared with each other on PGI Improvement. Figure S16. Funnel plot of the effect of 60 mg/d dose of duloxetine compared with placebo on SF-MPQ Total Score. Figure S17. Funnel plot of the effect of 120 mg/d dose of duloxetine compared with placebo on SF-MPQ Total Score. Figure S18. Funnel plot of the effect of both doses of duloxetine (60 mg/d and 120 mg/d) compared with each other on SF-MPQ Total Score. Figure S19. Funnel plot of the effect of 60 mg/d dose of duloxetine compared with placebo on Euro Quality of Life Questionnaire. Figure S20. Funnel plot of the effect of 120 mg/d dose of duloxetine compared with placebo on Euro Quality of Life Questionnaire. Figure S21. Funnel plot of the effect of both doses of duloxetine (60 mg/d and 120 mg/d) compared with each other on Euro Quality of Life Questionnaire. Figure S22. Funnel plot of the effect of 120 mg/d dose of duloxetine compared to routine care on Euro Quality of Life Questionnaire. Figure S23. Funnel plot of the effect of 60 mg/d dose of duloxetine compared with placebo on SF-36 Survey Bodily Pain. Figure S24. Funnel plot of the effect of 120 mg/d dose of duloxetine compared with placebo on SF-36 Survey Bodily Pain. Figure S25. Funnel plot of the effect of both doses of duloxetine (60 mg/d and 120 mg/d) compared with each other, on SF-36 Survey Bodily Pain. Figure S26. Funnel plot of the effect of 120 mg/d dose of duloxetine compared to routine care, on SF-36 Survey Bodily Pain. Figure S27. Funnel plot of the effect of 60 mg/d dose of duloxetine compared with doses of 300 to 900 mg/d of gabapentin, on VAS. Table S1. Excluded studies and the reasons for their exclusion. Table S2. PRISMA. Table S3. GRADE. Summary of Findings (SoF) and quality of evidence (GRADE) for Duloxetine in patients with neuropathic pain associated with Diabetes Mellitus.
